# Genetic Deletion of the Stromal Cell Marker CD248 (Endosialin) Protects against the Development of Renal Fibrosis

**DOI:** 10.1159/000438754

**Published:** 2015-12-04

**Authors:** Stuart William Smith, Adam Paul Croft, Hannah Louise Morris, Amy Jane Naylor, David Leonard Huso, Clare Marie Isacke, Caroline Olivia Susan Savage, Christopher Dominic Buckley

**Affiliations:** ^a^Center for Translational Inflammation Research, University of Birmingham Research Laboratories, Queen Elizabeth Hospital, London, UK; ^b^Wellcome Trust Clinical Research Facility, Queen Elizabeth Hospital Birmingham NHS Foundation Trust, Birmingham, London, UK; ^c^Breakthrough Breast Cancer Research Centre, The Institute of Cancer Research, London, UK; ^d^Department of Molecular and Comparative Pathobiology, The Sidney Kimmel Comprehensive Cancer Center, John Hopkins Medical Institutions, Baltimore, Md., USA

**Keywords:** CD248, Endosialin, Fibrosis, Kidney

## Abstract

**Background:**

Tissue fibrosis and microvascular rarefaction are hallmarks of progressive renal disease. CD248 is a transmembrane glycoprotein expressed by key effector cells within the stroma of fibrotic kidneys including pericytes, myofibroblasts and stromal fibroblasts. In human disease, increased expression of CD248 by stromal cells predicts progression to end-stage renal failure. We therefore, hypothesized that the genetic deletion of the *CD248* gene would protect against fibrosis following kidney injury.

**Methods:**

Using the unilateral ureteral obstruction (UUO) model of renal fibrosis, we investigated the effect of genetic deletion of *CD248* on post obstructive kidney fibrosis.

**Results:**

CD248 null mice were protected from fibrosis and microvascular rarefaction following UUO. Although the precise mechanism is not known, this may to be due to a stabilizing effect of pericytes with less migration and differentiation of pericytes toward a myofibroblast phenotype in CD248^-/-^ mice. CD248^-/-^ fibroblasts also proliferated less and deposited less collagen in vitro.

**Conclusion:**

These studies suggest that CD248 stromal cells have a pathogenic role in renal fibrosis and that targeting CD248 is effective at inhibiting both microvascular rarefaction and renal fibrosis through modulation of pericyte and stromal cell function.

## Introduction

Tissue fibrosis and microvascular rarefaction are the pathological hallmarks of progressive end-stage renal disease [1,2]. It has recently been established that resident microvascular pericytes and perivascular fibroblasts are the predominant source of the activated, matrix depositing stromal cell population in fibrotic kidney tissue [3,4,5]. However, there are currently no therapeutic strategies that directly target these disease processes. It has been established that pericyte activation leads to detachment from the vasculature, migration and differentiation into the scar-forming myofibroblasts within the tubulointerstitium. This leads to destabilization of vascular integrity, microvascular rarefaction and tissue hypoxia [6,7]. The histological evidence of these disease processes (vessel rarefaction and the extent of tubulointerstitial fibrosis) within kidney biopsy tissue is known to correlate with poor prognostic outcomes in patients with CKD [2,7,8]. In view of the key effector roles of pericytes, perivascular fibroblasts and myofibroblasts, identification and functional characterization of novel proteins expressed by these resident cell types will be critical for the therapeutic modulation of these disease pathways.

CD248 (endosialin) is a type I transmembrane glycoprotein that is expressed by stromal fibroblasts, myofibroblasts and a subset of pericytes, but not by endothelial cells [9,10,11,12]. Upregulated expression of CD248 has been observed following inflammation, and the constitutive loss of CD248 has been shown to modulate inflammatory tissue stroma [13,14,15]. The function of CD248 has not been fully elucidated; however, it has been shown to regulate platelet-derived growth factor (PDGF) receptor (PGDFR) signaling and bind fibronectin and collagens types I and IV [16]. Binding of these ligands is thought to mediate stromal cell adhesion, migration and proliferation in vitro [17].

We have previously demonstrated in kidney biopsy tissue that *CD248* is expressed by mesangial pericytes of the glomerulus, and expressed at low levels by a population of peritubular cells that morphologically resemble pericytes [10]. *CD248* expression was upregulated in stromal compartment of fibrotic kidneys being expressed by stromal fibroblasts and tissue myofibroblasts. The level of expression correlated with progression of renal disease. We thus hypothesised that *CD248*-expressing stromal cells are pathogenic in renal fibrosis. To test our hypothesis, we have induced renal fibrosis using the unilateral ureteral obstruction (UUO) model of kidney injury in CD248^-/-^ mice. We demonstrate that CD248^-/-^ mice are protected against myofibroblast accumulation, microvascular rarefaction and renal fibrosis.

## Materials and Methods

### Mice and Surgery

129SvEv mice were purchased from Taconic, Denmark and maintained in 12-hour light/12-hour dark cycle with free access to food and water. All procedures were performed in accordance with UK Home Office guidelines. Transgenic mice lacking CD248^-/-^ were generated and genotyped as previously described [18]. For all in vivo studies, 6 litter-matched male mice aged 8-10 weeks were used in each experimental group. Experiments were repeated 3 imes in independent experiments. Bone marrow chimeras were generated as previously described [19] using fetal liver cells expressing enhanced yellow fluorescent protein under the control of the Rosa26 promoter. Unilateral ureteric obstruction was performed by midline laparotomy; in brief, the left ureter was identified and ligated at 2 points. Sham-operated control mice underwent an identical procedure except that the left ureter was mobilized but not ligated. For all in vivo studies, 6 litter-matched male mice aged 8-10 weeks were used in each experimental group. Experiments were repeated 3 times in independent experiments.

### Immunohistochemistry

Four-micrometer tissue paraffin sections were dewaxed in xylene and re-hydrated. Antigen retrieval was performed using Dako Target Antigen Retrieval solution (Dako) at 95°C. Sections were stained according to the Dako rabbit Envision-HRP kit protocol. Sections were then counterstained with Mayer's hematoxylin. Rabbit anti-mouse CD248 unconjugated (clone PI3, in house antibody) was used to localize *CD248* expression [12]. Isotype controls were substituted for primary antibody on serial sections. Renal fibrosis was visualized and quantified with use of Picrosirius red stain. Twelve non-overlapping fields at ×400 magnification from each section were captured using a Nikon Eclipse E400 microscope and digital camera (Nikon). The threshold of pixels was expressed as a percentage of the total pixels using Adobe Photoshop. This was taken to represent the percentage area staining positively for collagen.

### Confocal Microscopy

The following were the primary antibodies: rat anti-mouse CD45 (eBioscience), rat anti-mouse PDGFRβ biotin (1:100; eBioscience, USA; clone APB5), anti-mouse alpha SMA (clone 1A4, Sigma), hamster anti-CD31 (1:100; Serotec, BioRad, USA; clone 2H8), anti-CD248 (1:400; clone p13, gift from Claire Isacke, The Institute of Cancer Research, London, UK), anti-NG2 (1:75; Upstate, Merck Millipore, USA; clone 132.38), mouse anti-desmin (Dako, 1:100). Primary antibodies were detected using appropriately conjugated secondary antibodies. DAPI was used as a nuclear counterstain.

For quantification, specific cells were counted in randomly selected fields of view at ×400 or ×630 magnification per mouse tissue section. Twenty-five random fields of view were analyzed per tissue section in 3 independent experiments. A cell was recorded as positive if 75% of the cell area immediately surrounding nuclei stained positive. A Zeiss confocal LSM 510 microscope (Zeiss, Germany) was used to visualize staining with images and captured and processed using the Zeiss LSM Image Examiner software (Zeiss). Representative images were chosen from each experiment for figure editing. For microvascular studies, quantification of the number of fluorescence pixels was calculated using Adobe Photoshop. The percentage area per field covered by vessels (positive CY-3 immunofluorescence) was then expressed as a percentage of the total pixel count. Results were expressed as mean ± SEM of 10 fields of view per mouse tissue section.

### Western Blotting

Tissue was homogenized in CellLyticMT (Sigma) sample buffer as per the manufacturer's instructions. Protein content was quantified using the Bradford assay (BioRad). Equivalent amounts of protein were loaded onto a 10% w/v non-denaturing SDS-PAGE gel. Gels were transferred onto PVDF membrane (GE Healthcare) and incubated with the primary antibodies: rabbit anti-mouse CD248 (clone PI3 [9]) or with anti-mouse GAPDH (Abcam) followed by horseradish peroxidase-conjugated anti-rabbit IgG (Amersham, Buckinghamshire, UK). Immunodetection was carried out using an enhanced chemiluminescence kit (Amersham, Buckinghamshire, UK) followed by exposure to X-ray film.

### Quantitative Polymerase Chain Reaction

RNA was extracted using an RNeasy kit (Qiagen) and transcribed into cDNA using a TaqMan® Reverse Transcription (Applied Biosystems) kit as per the manufacturer's instructions. Quantitative PCR was performed using TaqMan Gene Expression assays; *CD248* (Mm00547485), *Collagen 1A1* (Col1a1; Mm00801666) and *GAPDH* (4352339E) were used as a housekeeping gene. Assays were run on a 7900HT Real Time PCR System (Applied Biosystems). CT values were normalized using *GAPDH* as a housekeeping gene (ΔCT). Fold changes in gene expression were then calculated using 2^-ΔΔCT^.

### Isolation of Renal Cells

Renal cell populations were isolated from murine kidney using the Dynabead infusion method described by Takemoto et al. [20]. The kidneys were then harvested, minced and a digested with collagenase IV 10 mg/ml in Hank's buffer with 200 U/ml DNase I (Roche) for 30 min at 37°C. The glomeruli were then separated from the tubules using a magnet (Invitrogen). Glomeruli were plated in RPMI 1640 (Gibco) containing 20% fetal calf serum (Gibco), 1.75 mm glutamine, 87 U/ml penicillin, 87 μg/ml streptomycin, 15 mm HEPES and 1 mm sodium pyruvate (all from Gibco) for the outgrowth of mesangial cells; tubules were plated in DMEM/F12 (Gibco) containing 20% fetal calf serum, 1.75 mm glutamine, 87 U/ml penicillin, 87 μg/ml streptomycin for the outgrowth of renal fibroblasts or in DMEM/F12 (Gibco) containing 0.5% fetal calf serum, 1.75 mm glutamine, 87 U/ml penicillin, 87 μg/ml streptomycin, 10 μg/ml insulin, 5.5 μg/ml transferrin, 5 ng/ml sodium selenite, 1 ng/ml endothelial growth factor (Sigma UK), 5 pg/ml triiodothyronine, 5 μg/ml dexamethasone and 2.5 μg/ml amphotericin (all from Gibco) for the isolation of proximal tubular epithelial cells. Cell identity was confirmed by morphology and expression of cell-specific markers. All cell cultures were grown through multiple passages (passage 4) to ensure homogeneity. All cells were negative for CD45, CD31 and podocyte marker, synaptopodin. Mesangial pericytes and tubulointerstitial fibroblasts were positive for CD90, fibronectin and vimentin but negative for the epith.

### Functional Studies

Eight thousand cells were seeded per well in a 96-well plate (counted using a Beckman Coulter Counter). Cells were serum starved for 24 h (media replaced with complete media in the absence of serum). Serum media was replaced at 24 h and MTT (MTT 3-(4,5-dimethylthiazol-2-yl)-2,5-diphenyltetrazolium bromide) assay was then performed according to manufacturer's instructions (Life Technologies). Crystal violet staining was used in parallel to ensure that the increased MTT activity correlated with an increase in cell number. A Picrosirius red dye-binding assay originally described by Heng et al. [21] was used for measuring collagen accumulation in vitro.

### Statistical Analysis

Results are presented as means ± SEM. The statistical significance of differences between means was assessed using 1-way or 2-way analysis of variance (ANOVA) with appropriate post-test analysis or 2-tailed Student's t test. Values of p < 0.05 were considered significant. Analysis and graphs were prepared using Prism 5 (GraphPad, San Diego, Calif., USA).

## Results

### CD248 Is Expressed in Adult Murine Kidney by a Subset of Pericytes and Perivascular Fibroblasts and Upregulated in the Tissue Stroma of Inflamed Kidneys

**Fig. 1:**
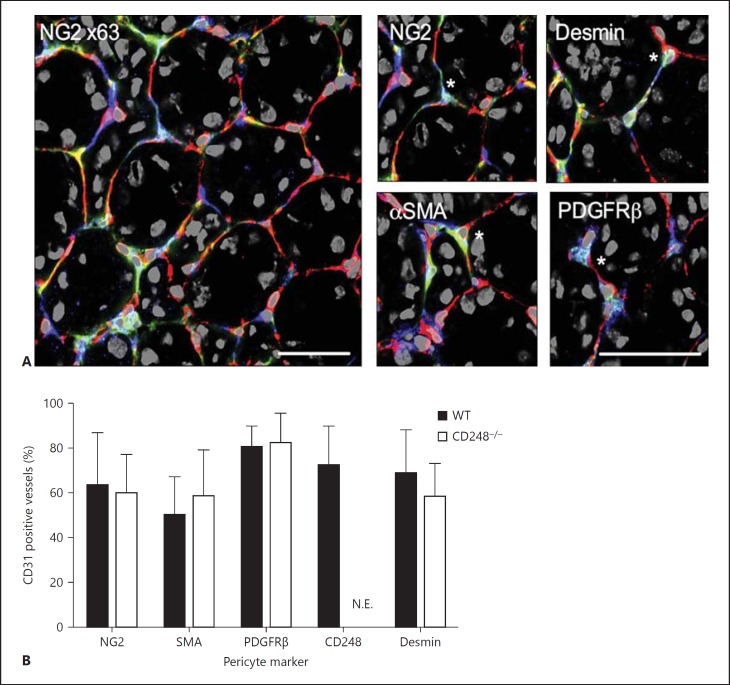
*CD248* is expressed by a subset of pericytes resting kidney. **A** CD248 co-localized with recognized markers of pericytes in resting kidney. Representative images are shown at ×630 magnification of WT resting kidney (right panel) with high-powered images (2 × zoom of ×630 original magnification) on the left panel stained with CD31 (endothelium, red), CD248 (blue) and pericyte markers: NG2, αSMA, PDGFRβ and desmin (green). Scale bar 50 μm. * Indicates an example of cell staining positive for both CD248 (blue) and the pericyte marker (green). **B** Expression of individual pericyte markers, expressed as percentage of CD31 positive vessels positive for PDGFRβ, NG2, αSMA or desmin. Filled bars are WT and open bars are CD248^-/-^. Data presented are mean ± SEM from 10 high-powered (×630) fields of view per mouse kidney section (10 × kidney sections were analyzed from 6 individual mouse). N.E. = Not expressed.

Consistent with our previous observations [10], we found CD248 was expressed by a subset of peritubular cells within the stromal compartment of resting murine kidney (fig. 1A). The close proximity of these cells to the vasculature suggested that *CD248* might be expressed by a subset of vascular pericytes. We identified pericytes histologically with a panel of antibodies against PDGFRβ, NG2, αSMA and desmin and expression was seen to co-localize with a subset of pericytes identified by these antibodies (fig. 1A). In kidney tissue from both wild type (WT) and CD248^-/-^ mice between 62 and 95% of CD31-positive vessels within the tissue were associated with pericyte cell bodies or processes (fig. 1B) depending on the pericyte marker that was used. The pericyte coverage of kidney endothelium is consistent with the observations of previous studies [22]. CD248 was visible on some of these pericytes and also occasionally on cells where none of the 3 other pericyte markers used were detectable (fig. 1). There were no significant differences between pericyte coverage (fig. 1B), or expression of each pericyte marker in tissue from CD248^-/-^ compared to WT mice (fig. 1B). As expected, *CD248* expression on pericytes was not expressed in CD248^-/-^ kidney tissue (fig. 1B).

**Fig. 2:**
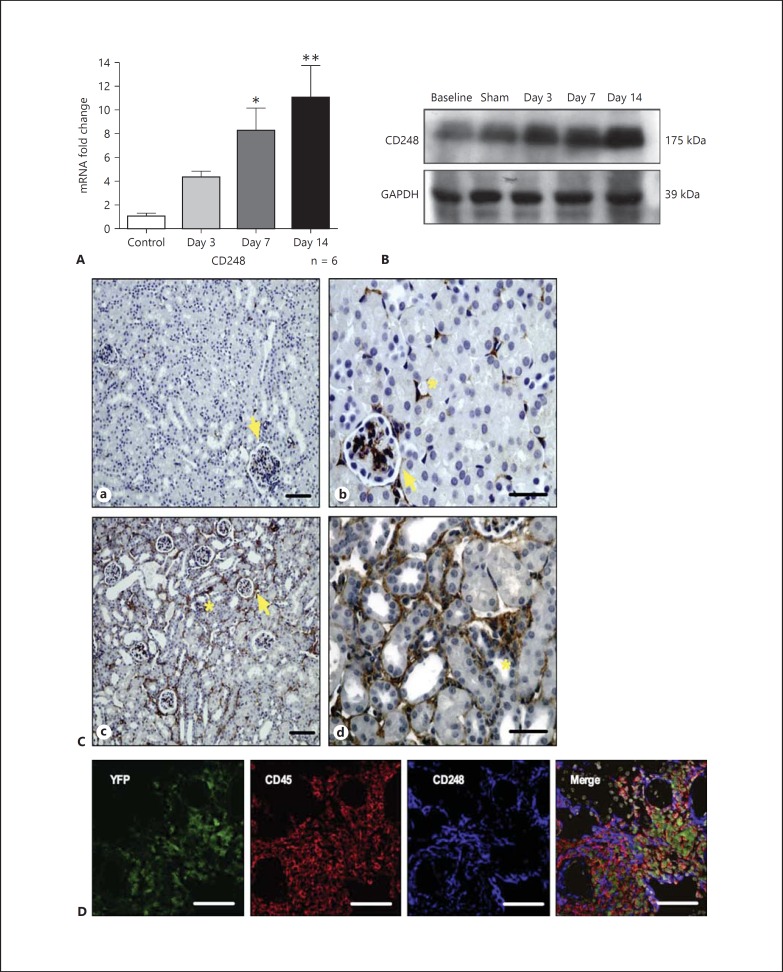
*CD248* expression increases following unilateral ureteric obstruction. Expression of CD248 was examined by quantitative PCR on whole kidneys (**A**). Results are expressed as mRNA fold change mean ± SEM relative to the control group (sham operated day 14; 1-way ANOVA with Bonferroni's post test relative to control, * p < 0.01, ** p < 0.001, n = 6 mice per treatment group). **B** Whole kidney lysates were also used to demonstrate CD248 protein expression at baseline (no operation performed), following a sham operation on day 14 and after UUO for 3, 7 and 14 days. **Ca**, **b** Tissue histology of sham-operated kidney tissue showed *CD248* was expressed by mesangial cells of the glomerulus (arrow) and by peritubular cells (*). At 14 days following UUO, tubular dilatation and interstitial expansion accompanied increased *CD248* expression in the interstitium (*) in a space expected to be occupied by pericytes and stromal fibroblasts (**Cc**, **d**). Magnification ×20 (**Ca**, **c**) and ×63 (**Cb**, **d**). **D** Bone marrow chimera studies. UUO-injured tissue from day 14 is shown. Yellow fluorescent protein (green) is expressed in the cytoplasm of infiltrating cells many of which are CD45+ leucocytes (red) but not within resident CD248+ stromal cells (blue). Magnification ×40 (representative images from n = 8 chimeric mice generated). Scale bar 50 μm.

Renal fibrosis was induced using the UUO model of renal fibrosis as previously described [23]. In this model, ligation of the ureter leads to an obstructive nephropathy and progressive tissue fibrosis. Kidney tissue was harvested and digested at 3, 7 and 14 days following UUO and from sham-operated mice. Following UUO, CD248 mRNA expression was found to be significantly increased at 7 and 14 days post UUO (fig. 2A), and this increased expression was also confirmed at the protein level (fig. 2B). Using immunohistochemistry, we observed that the upregulated expression of *CD248* occurred within the stromal compartment of the kidney with CD248 being highly expressed by vascular pericytes and stromal fibroblasts/myofibroblasts (fig. 2C). In contrast, in sham-operated kidney tissue, *CD248* expression was low and restricted to a small number of vascular pericytes and mesangial cells of the glomerulus. We next performed bone marrow chimera studies to confirm that following UUO, CD248 was expressed only by resident stromal cells and not by infiltrating (donor derived) bone marrow-derived cells (fig. 2D).

### CD248^*-/-*^ Mice Are Protected against the Development of Renal Fibrosis Following UUO

To examine the role of CD248 in renal fibrosis, we performed UUO in CD248^-/-^ mice. At baseline, there was no significant difference in body weight, urinary protein loss, renal function or the histological appearance of the tubulointerstitial space and glomerular compartments between WT and CD248^-/-^ mice (p > 0.05, ns, n = 10 mice per group, data not shown). Following UUO, the severity of renal fibrosis was determined by histological examination for collagen deposition using Sirius red stain. There was a significant increase in the deposition of fibrillary collagen in the tubulointerstitial space 7 and 14 days post UUO (*** p ≤ 0.001) compared to sham-operated mice in both WT and CD248^-/-^ mice. However, CD248^-/-^ mice deposited significantly less collagen after 14 days of UUO (42% reduction, ** p < 0.01) compared to WT mice. Similarly, collagen 1a1 (Col1a1) mRNA transcription was significantly increased 7 and 14 days following injury compared to sham-operated tissue. In CD248^-/-^ mice, col1a1 RNA expression was increased in response to injury (* p < 0.01) but levels were blunted compared to WT mice, and there was no significant difference in expression at days 3, 7 and 14 post UUO. Col1a1 RNA expression was significantly reduced in CD248^-/-^ mice at day 14 following injury compared to WT mice (** p < 0.01).

**Fig. 3:**
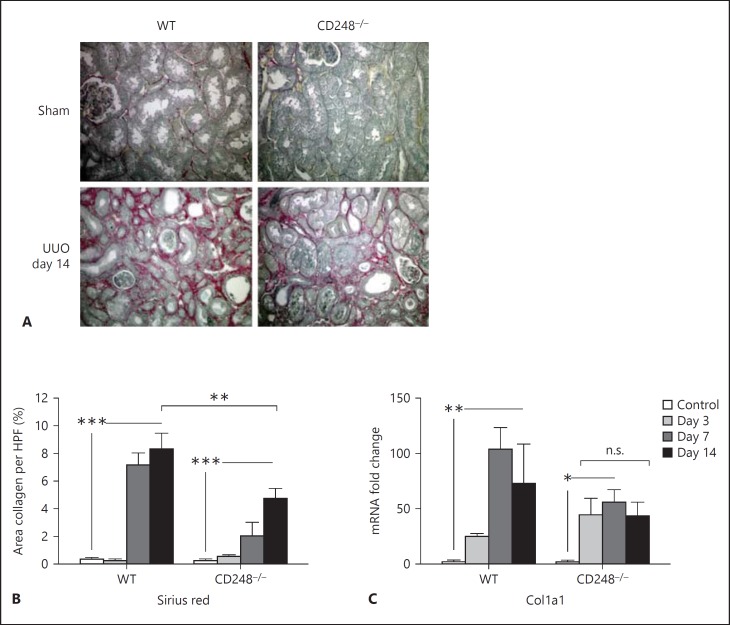

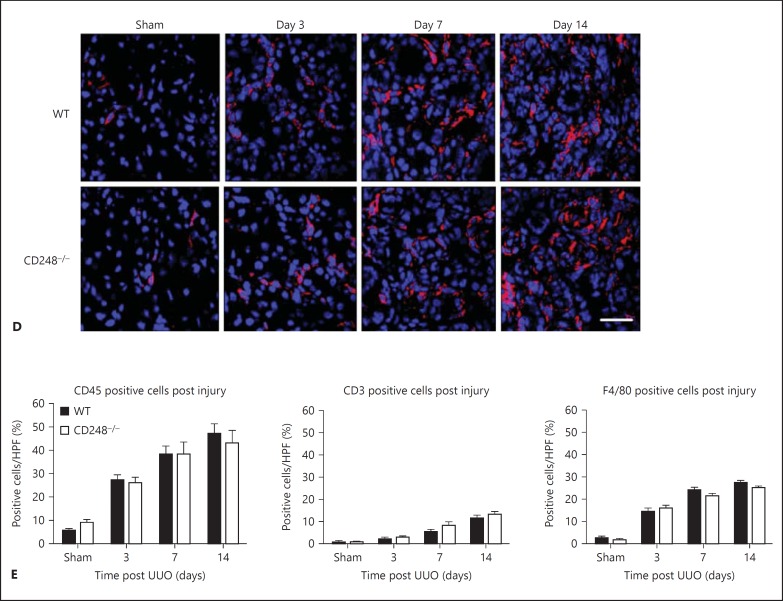
CD248^-/-^ mice accumulate less interstitial collagen than WT mice following unilateral ureteric obstruction despite no significant difference in leukocyte infiltration. **A** Picrosirius red staining of sham-operated and UUO day 14 kidney tissue from WT and CD248^-/-^ mice (×40 magnification). **B** Digital quantification of Picrosirius red staining expressed as the percentage area of collagen per high power field. Collagen (red) expression is increased in WT and CD248^-/-^ mice following injury. CD248^-/-^ mice deposited significantly less collagen after 14 days of UUO (42% reduction, 2-way ANOVA, n = 6, *** p < 0.001, ** p < 0.01). **C** Col1a1 RNA expression was measured by quantitative PCR on whole kidneys. Results are expressed as mRNA fold change mean ± SEM relative to the control group (sham operated day 14). Col1a1 RNA expression was significantly reduced in CD248^-/-^ mice at day 14 following injury compared expression in WT mice (2-way ANOVA, n = 6, * p < 0.05, ** p < 0.01). **D** Representative confocal images of leucocyte infiltration defined by the expression of CD45 (red), nuclear stain (blue) in kidney tissue sections from WT and CD248^-/-^ mice undergoing UUO. Magnification ×40. Scale bar 50 μm. **E** Digital quantification of CD45, CD3 and F4/80 expression in kidney tissue sections from WT and CD248^-/-^ mice following UUO. Data are expressed as mean ± SEM percentage of positive cells in 10 random fields of view per kidney section at a magnification of ×40 in 3 independent experiments (total n = 30 images analyzed). No significant difference in leucocyte infiltration was observed between WT and CD248^-/-^ mice.

To determine if CD248^-/-^ mice were protected from renal fibrosis as a result of a change in leucocyte accumulation within the tissue, we examined the numbers of CD45, CD3 and F4/80 expressing leucocytes in kidney tissue using confocal microscopy. Following UUO, there was a progressive infiltration of leukocytes in fibrotic kidney tissue but no significant difference in the number of infiltrating leucocyte populations in CD248^-/-^ mice compared to WT mice (fig. 3E).

### Pericyte Attachment in Response to Renal Injury

**Fig. 4:**
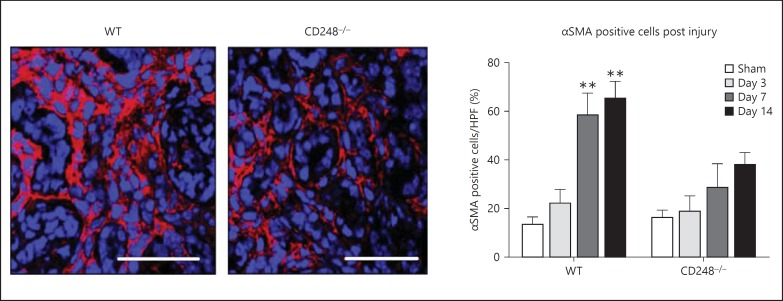
CD248^-/-^ mice have fewer αSMA+ myofibroblasts. Images showing αSMA expression (red) in injured kidney (14 days post UUO) in both WT and CD248^-/-^ cells. Nuclei blue. Magnification ×630. Scale bar 50 μm. In WT mice, there was a significant increase in the number of αSMA+ cells at 7 and 14 days following injury compared to sham-operated mice (1-way ANOVA and post hoc, n = 6 mice per time point, ** p < 0.001). In contrast, the increase in αSMA+ cells was not observed in CD248^-/-^ mice.

Vascular pericytes have been shown to migrate into the kidney tissue stroma and differentiate into myofibroblasts following injury [6]. As *CD248* was found to be highly expressed by a subpopulation of pericytes during inflammation, we next determined whether CD248^-/-^ mice were protected from renal fibrosis as a result of having less tissue myofibroblasts. Following renal injury in WT mice, the number of interstitial myofibroblasts as marked by αSMA was significantly increased 7 and 14 days post UUO compared to sham-operated mice (** p ≤ 0.001). In contrast, there was no statistically significant increase in αSMA cells in following UUO in CD248^-/-^ mice compared to sham-operated animals (fig. 4).

**Fig. 5:**
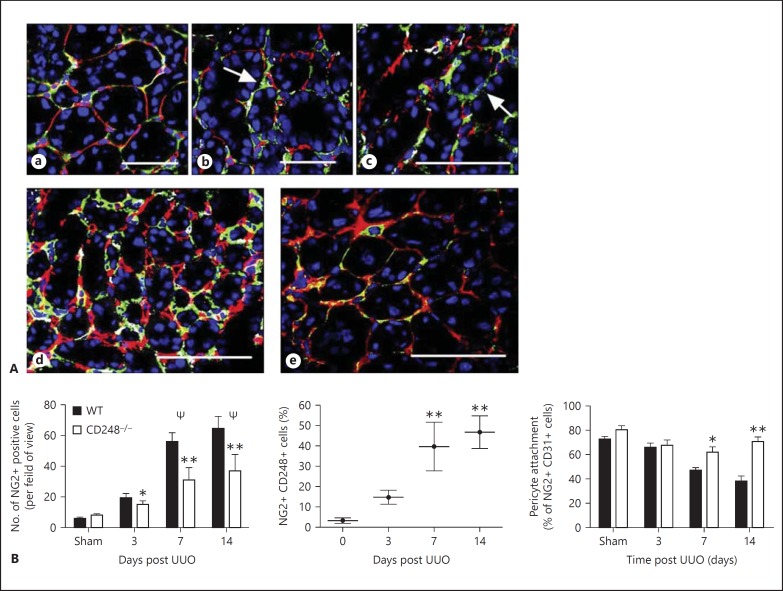
Less pericytes are seen in association with endothelium and in the kidney interstitium in CD248^-/-^ mice following UUO. Representative immunostaining for CD31 (red), CD248 (white), NG2 (green) and nuclei blue (**A**) from sham-operated mice (**Aa**), WT mice 7 days (**Ab**) and 14 days (**Ac**) post UUO ×630 magnification. White arrows indicate pericytes not closely associated with endothelium. WT (**Ad**) and CD248^-/-^ (**Ae**) kidney tissue 14 days post UUO (×400 magnification). **B** Quantification of NG2 positive fibroblasts in WT vs. CD248^-/-^ mice and NG2+ CD248+ pericytes in WT kidney tissue following injury. Data are presented as mean ± SEM for percentage of positive cells per field of view (10 random fields of view at ×400 magnification selected per treatment group in 3 independent experiments, n = 6 mice per treatment group). The percentage of cells expressing NG2 in kidney tissue of both WT and CD248^-/-^ mice was significantly increased 3 days (* p = 0.05), 7 and 14 days following UUO (Ψ p ≤ 0.01). The percentage of NG2+ cells in WT mice was significantly (** p ≤ 0.01) increased in kidney tissue from WT vs. CD248^-/-^ mice at 7 and 14 days post UUO. The percentage of NG2 cells attached to endothelium (defined as co-localization with CD31) was also quantified. In WT mice the percentage of NG2+ CD248+ were significantly increased (** p ≤ 0.01) 7 and 14 days post injury compared to sham-operated mice. There was a significant increase in the number of pericytes (NG2-expressing cells) attached to the endothelium at 7 and 14 days post UUO in CD248^-/-^ mice compared to WT mice (* p ≤ 0.01, ** p ≤ 0.05).

We next determined whether the reduction in myofibroblast number correlated with a change in the numbers of vascular pericytes in the kidneys of CD248^-/-^ mice following UUO. NG2 staining was used to identify vascular pericytes, and attachment of pericytes to endothelium was defined by adjacent staining of the pericyte marker with CD31 (>50% cell contact). In sham-operated mice, kidney pericytes were found in association with 62 (±23%) of blood vessels (fig. 5Aa) but following injury, less pericytes were seen in close apposition with endothelial cells at day 7 (fig. 5Ab) and day 14 (fig. 5Ac) following UUO. In WT mice 14 days post UUO, the percentage of pericytes in the stromal tissue increased, and less pericytes were seen closely attached to endothelium (fig. 5Ad). In comparison, the percentage of pericytes was less in kidney tissue from CD248^-/-^ mice 14 days following UUO and most were seen in close association with endothelium (fig. 5Ae).

These findings were confirmed by quantification of pericyte number and the number of pericytes in close association with CD31+ cells in WT and CD248^-/-^ kidney tissue following UUO. Following UUO, the number of NG2-positive pericytes were significantly increased in both WT and CD248^-/-^ mice at days 3 (* p ≤ 0.05), 7 and 14 (Ψ p ≤ 0.01) post injury compared to sham-operated mice (fig. 5B). However, the magnitude of the increase in pericytes was significantly less in CD248^-/-^ mice. At day 3, there was no significant difference in the percentage of NG2-positive cells between WT and CD248^-/-^ mice but at days 7 and 14, the number of NG2-positive cells was significantly greater in WT mice compared to CD248^-/-^ mice (p ≤ 0.01). In WT mice, there was a specific expansion in CD248+ NG2+ cells 7 days (** p ≤ 0.01) and 14 days (** p ≤ 0.01) post UUO compared to sham-operated mice. Finally, the number of pericytes in close association with endothelium (defined by >50% of pericyte in contact with CD31+ cells) was significantly reduced at day 7 (* p ≤ 0.05) and day 14 (** p ≤ 0.01) post UUO in WT mice compared to sham-operated mice. In contrast, no significant difference was observed in CD248^-/-^ mice post UUO compared to sham-operated mice.

### CD248^*-/-*^ Mice Are Protected from Microvascular Rarefaction Following UUO

**Fig. 6:**
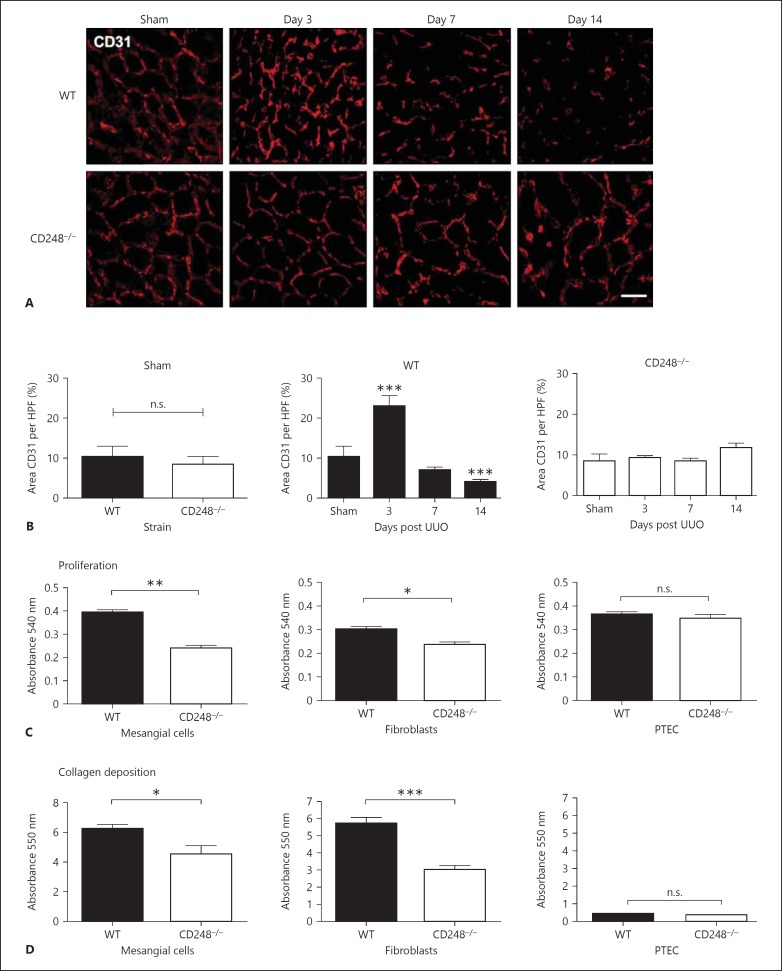
Microvascular rarefaction is reduced in CD248^-/-^ mice. **A** and **B** CD31+ staining in UUO kidneys from WT and CD248^-/-^ mice. Vessel density was assessed at various time points following UUO in sham-operated, WT and CD248^-/-^ mice (1-way ANOVA, n = 6 mice per group at each time point repeated in 3 independent experiments, * p < 0.05, *** p < 0.001 compared to control). **C** and **D** The proliferative capacity and ability to deposit collagen of stromal cells (fibroblasts and mesangial cells) and epithelial cells from WT and CD248^-/-^ animals were assessed. Stromal cells but not epithelial cells from CD248^-/-^ mice demonstrated a reduced capacity to proliferate and deposit collagen in vitro (t test, n = 3, * p < 0.05, *** p < 0.01).

Prior to injury, there was no difference in vessel area between WT and CD248^-/-^ animals (fig. 6B). Following injury, there was an initial increase in vessel density in WT mice 3 days post injury followed by progressive vascular regression 14 days post injury (fig. 6B) suggesting an early phase of reparative angiogenesis followed by vascular regression (microvascular rarefaction), as previously reported [6]. In contrast, CD248^-/-^ mice did not have an early phase of reparative angiogenesis and were protected from microvascular rarefaction in response to injury as no significant difference in CD31 area could be detected between sham-operated animals and day 14 injured mice (fig. 6B far right panel).

### CD248^*-/-*^ Stromal Cells Exhibit Impaired Function in vitro

In order to determine whether the effects we observed in vivo were due to a direct effect of *CD248* expression on stromal compartment in the kidney, we isolated stromal fibroblasts, mesangial cells, both of which express *CD248*, and as a control, we isolated proximal tubular epithelial cells, which do not express *CD248*. These cells were isolated from WT and CD248^-/-^ mice. The primary cells were isolated and the homogeneity of cell cultures confirmed as described in the methods. Fibroblasts and mesangial cells, but not proximal tubular epithelial cells isolated from CD248^-/-^ mouse kidneys exhibited a significantly reduced proliferative potential compared to cells isolated from WT mice (fig. 6C). Similarly, fibroblasts and mesangial cells, but not proximal tubular epithelial cells isolated from CD248^-/-^ mice exhibited a reduced capacity to deposit collagen in vitro (fig. 6D). These results suggest that the absence of CD248 can have a direct effect on the function of stromal cells in vitro.

## Discussion

In the present study, we demonstrate a pathogenic role for CD248 in the development of renal fibrosis. We show that *CD248*-deficient mice are protected from renal fibrosis and microvascular rarefaction. These observations build on our previous work showing an upregulation of CD248 within the kidney biopsies of patients with progressive fibrotic renal disease. Collectively our findings suggest that targeting CD248 may be an effective therapeutic strategy to inhibit tissue fibrosis and microvascular rarefaction through modulation of both pericyte and stromal cell function.

Whilst the exact mechanism by which *CD248*-expressing stromal cells contribute to fibrosis is not yet fully known, we found that in the absence of CD248, more vascular pericytes remain associated with the vascular endothelium and fewer matrix depositing myofibroblasts are seen in tubulointerstitium. These observations are consistent with recent reports establishing that the primary source of matrix depositing stromal cells are pericytes that migrate from the endothelium to the stromal compartment resulting in vascular instability [6]. Our findings suggest that *CD248* expression on stromal cells and pericytes is a critical step in regulating the effective operation of these 2 disease processes.

We have previously demonstrated that in resting kidney, *CD248* is expressed by mesangial pericytes of the glomerulus and at low levels by a population of peritubular cells that morphologically appeared to represent pericytes [10]. With progressive renal fibrosis, upregulated expression of *CD248* was observed within the stromal cell compartment of the kidney. In the murine kidney, *CD248* is expressed by mesangial cells of the glomerulus, stromal fibroblasts and some, but not all, pericytes surrounding the capillaries. PDGFRβ, NG2, αSMA and desmin are all widely used as pericyte markers, but none are specific for pericytes, nor expressed on all pericytes at all times [24]. While CD248 sometimes co-localized with PDGFRβ, NG2 and desmin, CD31-positive capillaries were also seen closely surrounded by CD248 single-positive perivascular cells, in a location expected to be occupied by pericytes. This suggests that CD248 can be useful in identifying a pericyte subset not detected by traditional pericyte markers. We have recently reported similar findings in skeletal muscle where *CD248* is expressed by a subset of pericytes expressing traditional pericyte markers and a subset expressing only *CD248*[25]. Understanding the function and phenotype of these *CD248*-expressing pericytes is the subject of ongoing work.

In human disease, fibrosis and vascular rarefaction have consistently been reported to associate with a poorer disease outcome [7]. Renal tubulointerstitial pericytes have been implicated in the development of renal fibrosis [2,3,6]. Careful tracking and kinetic modeling studies performed by Lin et al. [6] have demonstrated that in response to injury, Col1a1+ pericytes, which express PDGFRβ and αSMA, detach and migrate away from the underlying endothelium to form myofibroblasts that deposit collagen. This process is accompanied by microvascular rarefaction with loss of pericytes from the vasculature and a failure of reparative angiogenesis [6]. In addition, pericyte proliferation has been shown to be associated with both ischemic-reperfusion and UUO mouse models of renal fibrosis [6,26]. However, we do not yet know if the reduced number of myofibroblasts and pericytes in the stroma in *CD248*-deficient mice is the result of impaired migration or a specific defect in proliferation in vivo.

In the UUO model, an early angiogenic response to injury has been demonstrated with increased capillary density detected from day 2 (inflammatory angiogenesis) followed by microvascular rarefaction [6]. In the absence of CD248, we observed that pericytes remain associated with the vascular endothelium and these kidneys are protected from vessel loss in response to injury. There is also blunting of the early angiogenic response to injury (lack of increase in vessel density at day 3). CD248-deficient mice also developed less stromal fibrosis following UUO. It has been demonstrated in cancer studies that CD248 expression is associated with tumor progression and metastasis [27]. CD248 binds to extracellular matrix proteins fibronectin, collagens types I and IV, and it is thought that the interaction between these components and CD248 mediates cell attachment and migration during tumor progression [17,28]. In the kidney, the absence of CD248 may prevent the interaction between pericytes, ECM proteins and stromal fibroblasts and prevent these cells from migrating from the vasculature into the tissue stroma and differentiating into myofibroblasts. This in turn would result in less stromal fibrosis following renal injury and stabilization of the vasculature preventing microvascular refraction.

PDGF signaling from endothelial cells to PDGFR on pericytes has been shown to be vital for vascular stabilization following kidney injury [22]. Recent evidence suggests that CD248 may exert its effects through regulation of the PDGF pathway as PDGF-induced phosphorylation of extracellular signal-regulated kinase (ERK), but not phosphorylation of PDGFR itself, was markedly diminished in CD248-deficient pericytes [16]. Thus, CD248 may modify PDGF signaling acting downstream of PDGFR but upstream of ERK1/2 by an as yet unknown mechanism. It is therefore possible that our observation that the absence of CD248 leads to less microvascular rarefaction and fibrosis following injury may be the result of defective PDGF pericyte signaling leading to decreased detachment from the endothelium in response to injury.

Renal injury and inflammation following UUO result in increased expression of CD248 on fibroblasts within the kidney stroma. In resting kidney, these cells did not express *CD248*. This increased expression of *CD248* was seen in addition to the increased subset of pericytes expressing *CD248* following UUO. The high expression of *CD248* in stromal cells in inflamed tissue, with low expression in healthy tissue makes it is an appealing therapeutic target. Targeting single receptors on pericytes such as PDGFRβ is sufficient in some models to prevent fibrosis [6,22]. However, pericyte detachment occurs early in the evolution of renal injury [5,] and therefore, blockade of CD248, which would lead to less pericyte detachment, is an attractive therapeutic candidate.

Our findings suggest that the constitutive removal of CD248 modulates the response of renal pericytes and stromal fibroblasts to injury. Although the precise mechanism is not yet known, it is clear that genetic loss of CD248 significantly reduces the formation of matrix depositing myofibroblasts in response to renal injury by ureteric obstruction, resulting in less tissue fibrosis. Our data highlight CD248 as a novel, anti-fibrotic, target with the added advantage that it may allow for the modulation of both activated pericytes and fibroblasts but not epithelial cells found in inflamed but not normal tissue. Targeting CD248 expressing stromal cells has the capability to modulate both microvascular rarefaction and tissue fibrosis, 2 vital disease processes in progressive renal fibrosis.

## Funding

This work was funded by a Wellcome Trust Training fellowship to Dr. Smith.

## Disclosure Statement

Professor C.O.S. Savage is currently employed by GlaxoSmith Kline and has also received research grants from Talecis and Biogen Idec. The other authors have nothing to disclose.
